# Analysis of the heteroplasmy level and transmitted features in hearing-loss pedigrees with mitochondrial 12S rRNA A1555G mutation

**DOI:** 10.1186/1471-2156-15-26

**Published:** 2014-02-17

**Authors:** Yuhua Zhu, Shasha Huang, Dongyang Kang, Mingyu Han, Guojian Wang, Yongyi Yuan, Yu Su, Huijun Yuan, Suoqiang Zhai, Pu Dai

**Affiliations:** 1 Department of Otorhinolaryngology, Head and Neck Surgery, PLA General Hospital, 28# Fuxing Road, Beijing 100853, P. R. China; 2 Department of Otolaryngology, Hainan Branch of PLA General Hospital, Haitang Bay, Sanya 572000, P. R. China

**Keywords:** Hearing loss, Mitochondrial DNA, Heteroplasmy, A1555G mutation

## Abstract

**Background:**

Mitochondrial cytopathies are characterized by a large variability of clinical phenotypes and severity. The amount of mutant mitochondrial DNA (mtDNA) in a cell, called the heteroplasmy level, is an important determinant of the degree of mitochondrial dysfunction and therefore disease severity. Understanding the distribution of heteroplasmy levels across a group of offspring is an important step in understanding the inheritance of diseases. Recently, the mtDNA A1555G mutation was found to be associated with non-syndromic and drug-induced hearing loss.

**Results:**

Here, we report five pedigrees with multiple members having the A1555G mutation and showing diverse clinical manifestations and different heteroplasmy levels. Clinical evaluations revealed that the hearing impairment phenotypes varied with respect to the severity of hearing loss, age of onset of hearing loss, and pattern of audiometric configuration. These five Chinese pedigrees had different penetrance of hearing loss, ranging from 10–52%. A molecular study showed that the average heteroplasmy rates of the five pedigrees were 31.98% (0–91.35%), 78.28% (32.8–96.08%), 87.99% (82.32–94.65%), 93.34% (91.02–95.05%), and 93.57% (91.38–94.24%). There was no gradual tendency of heteroplasmy to increase or decrease along with transmission. A study of the relationship between clinical features and genetic background found that the percentage of deafness was 0 when the heteroplasmy level was less than 50%, 25% when the heteroplasmy level was 50–80%, 47.06% when the heteroplasmy level was 80–90%, and 57.58% when the heteroplasmy level exceeded 90%. The risk of deafness rose with the heteroplasmy level.

**Conclusions:**

The results suggest that there are large random shifts in the heteroplasmy level between mothers and offspring with the A1555G mutation; heteroplasmy could disappear randomly when the heteroplasmy level of the pedigree was low enough, and no regular pattern was found. The heteroplasmy level may be one of the factors influencing the penetrance of deafness caused by the mtDNA A1555G mutation.

## Background

The mitochondrial 12S ribosomal RNA (rRNA) gene is a hotspot for mutations associated with aminoglycoside-induced, non-syndromic hearing loss. Of these mutations, the A1555G mutation at a highly conserved decoding region of the 12S rRNA has been reported to be associated with hearing loss in many ethnic populations [1-4]. In these pedigrees and individuals, the variable clinical phenotype and incomplete penetrance of the A1555G-induced hearing loss complicates our understanding of this mutation. Many factors could be involved in the variable phenotype of the A1555G mutation; these include ethnic background, environmental influences, aminoglycoside used, nuclear genes, mitochondrial haplotypes/variants, and a possible threshold effect [5-9]. For example, the mitochondrial tRNA variants tRNA^Glu^A14693G, tRNA^Arg^T10454C, tRNA^Thr^T15908C, and tRNA^Ser(UCN)^G7444A may contribute to a higher penetrance of hearing loss in pedigrees carrying the A1555G mutation [10,11]. In addition, as one of the most important hereditary features, the heteroplasmy level of a mitochondrial DNA (mtDNA) mutation may be another influential factor [12].

The mtDNA is inherited maternally. Heteroplasmy describes a condition in which cells containing mtDNA with pathogenic mutations also contain the normal wild-type mtDNA. The relative amount of mutant mtDNA compared with the wild-type mtDNA in a cell, called the heteroplasmy level, is an important determinant of the degree of mitochondrial dysfunction and therefore the disease severity. The risk for transmission of a mitochondrial disorder is difficult to estimate because of heteroplasmy. Therefore, elucidating the distribution of heteroplasmy levels across a group of offspring is an important step in understanding the inheritance of diseases caused by mtDNA mutations [13,14].

Here, we analyzed the clinical and genetic features of five heteroplasmy pedigrees with aminoglycoside-induced, non-syndromic hearing loss and the A1555G mutation, identified through a nationwide epidemiological survey, to explore the influence of heteroplasmy on the phenotype and inheritance of the A1555G mutation.

## Methods

### Subjects and audiological examinations

**Figure 1:**
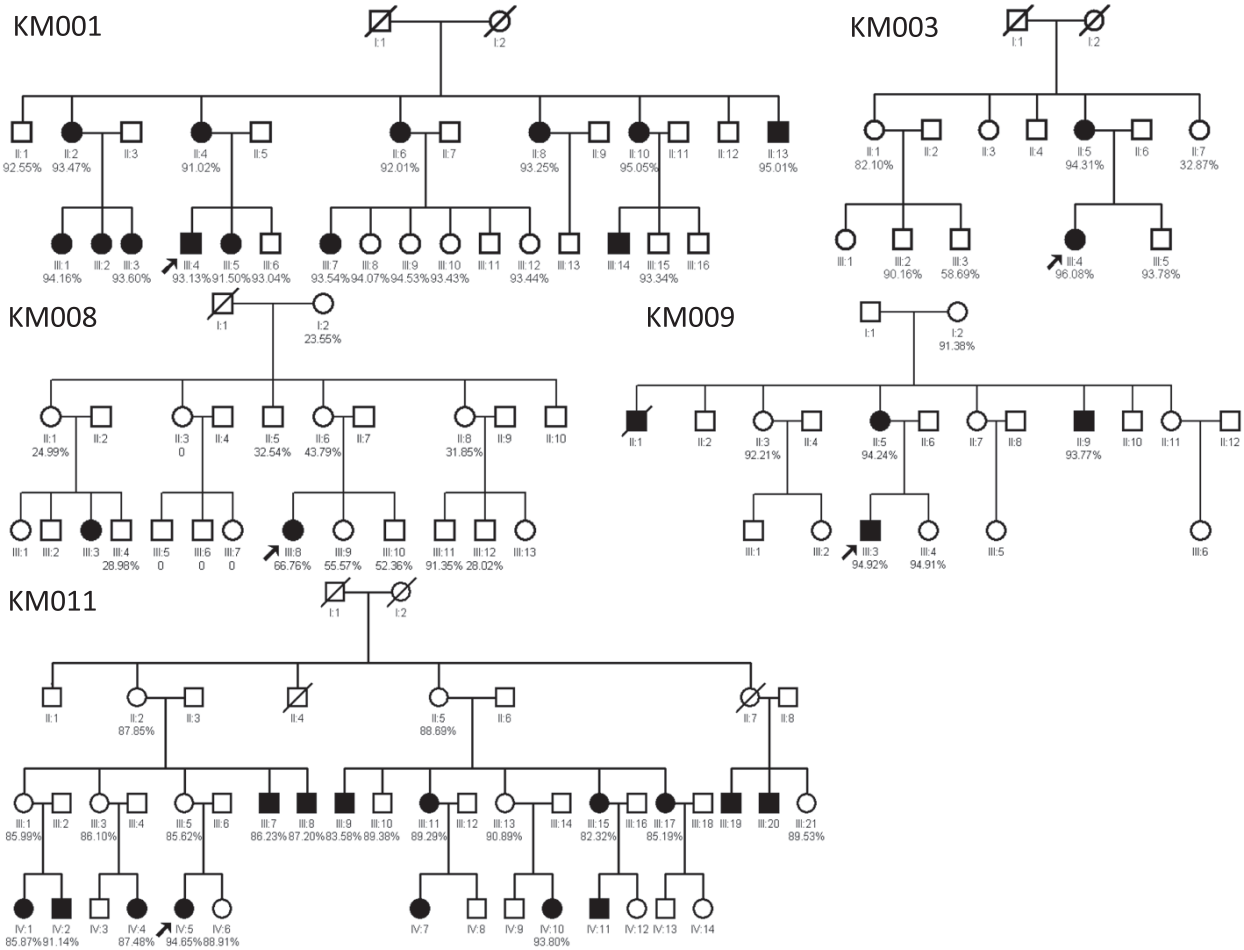
**Five Chinese pedigrees with aminoglycoside-induced and non-syndromic hearing impairment.** Hearing-impaired individuals are indicated by filled symbols. Arrow denotes probands. The heteroplasmy level is marked below the identifiers.

As part of a genetic screening program for hearing impairment, five Han Chinese families with the 12S rRNA A1555G mutation were identified by the Department of Otolaryngology of the Chinese PLA General Hospital, using mitochondrial 12S rRNA sequence analysis (Figure 1). A comprehensive history and physical examination were used to identify any syndromic finding, a history of aminoglycoside use, and genetic factors related to the hearing impairment in the pedigree members. Age-appropriate audiological examinations, including pure-tone audiometry (PTA) or auditory brainstem response, immittance testing, and distortion product otoacoustic emissions, were performed. The PTA was calculated from the sum of the audiometric thresholds at 500, 1000, 2000, and 4000 Hz. The severity of hearing impairment was classified into five grades: normal, <26 decibels (dB); mild, 26–40 dB; moderate, 41–70 dB; severe, 71–90 dB; and profound, >90 dB (Van Camp G. Hereditary Hearing Loss Homepage). Informed consent was obtained from the participants before their participation in the study, in accordance with the Ethics Committee of the Chinese PLA General Hospital. The pedigree maps were constructed using Cyrillic software ver. 2.1.3.1. The penetrance was calculated by dividing the affected number of matrilineal relatives by the total number of matrilineal relatives.

### Molecular study

#### *Direct sequencing*

Genomic DNA was isolated from the whole blood of participants. The subjects’ DNA spanning the entire mitochondrial 12S rRNA gene was amplified by PCR [15]. All genotypes were detected by direct sequencing and verified by forward and reverse sequencing using an ABI 3730 automated DNA sequencer (Applied Biosystems, Foster City, CA, USA). The resultant sequence data were compared with the updated consensus Cambridge sequence (GenBank accession number: NC_012920). The mtDNA A1555G mutation was identified in peripheral blood leukocytes in every patient.

#### *SNaPshot technique*

The level of heteroplasmy in peripheral blood leukocytes was determined using SNaPshot technology (Applied Biosystems) to estimate the areas of the peaks of the wild-type (A allele peak) and mutant (G allele peak) alleles at the 1555 site. The heteroplasmy rate was calculated as the area of the G allele/(area of the A allele + area of the G allele). This technique involves PCR amplification of the region of interest, purification of the product, and annealing of a SNaPshot primer that ends one nucleotide 5′ from a known single nucleotide polymorphism (SNP). A single base extension reaction is then performed in the presence of the four fluorescently labeled dideoxynucleotide triphosphates (ddNTPs). Upon excitation with a laser, each different fluorescent dye emits a color specific for a ddNTP, i.e., green for A, blue for G, black for C, and red for T.

A pair of PCR primers (forward, 5′-CCACCTCTTGCTCAGCCTAT-3′; and reverse, 5′-TAGCTCAGAGCGGTCAAGTT-3′) and a SNaPshot probe (5′-TTTTTTTTTTTTTTTTTTTTTTTTTTTCCCTACGCATTTATATAGAGGAG-3′) were designed. The resulting PCR fragment was 436 bp in size. The PCR reactions (Applied Biosystems) were performed in 40-μl volumes according to the following conditions: initial denaturation at 95°C for 2 min, five cycles of 94°C for 20 s, 55°C for 30 s, and 72°C for 40 s, and then five cycles of 94°C for 20 s, 53°C for 30 s, and 72°C for 40 s, followed by 25 cycles of 94°C for 30 s, 52°C for 30 s, and 72°C for 40 s, with a final 5-min extension at 72°C. After the PCR, SNaPshot extension reactions were carried out on an ABI PRISM 3730 DNA sequencer (Applied Biosystems). All samples were genotyped using GeneMapper software.

#### *Mutational analysis of GJB2 gene*

The DNA fragments spanning the entire coding region of GJB2 gene were amplified by PCR using the following oligodeoxy-nucleotides: forward-5′TATGACACTCCCCAGCACAG’ and reverse-5′GGGCAATGCTTAAACTGGC3′. PCR amplification and subsequent sequencing analysis were performed as detailed elsewhere. The results were compared with the wild type GJB2 sequence (GenBank accession number: M86849) to identify the mutations.

## Results

### Clinical characteristics of the hearing-loss patients in five pedigrees

**Figure 2:**
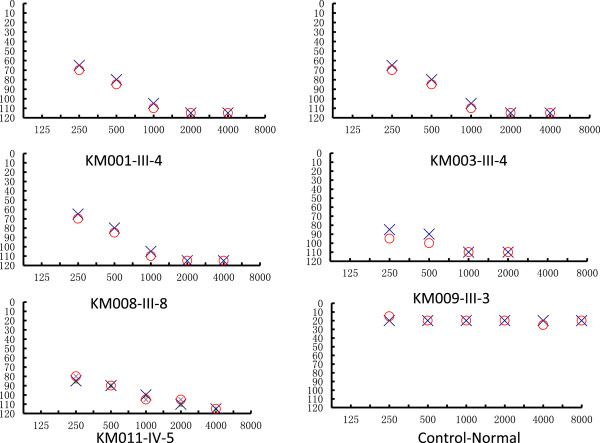
**Air conduction audiogram of 5 affected probands with the A1555G mutation and one Chinese control.** Symbols: X—left ear, O—right ear.

A comprehensive history and physical and audiological examinations were performed to identify syndromic phenotypes. The history of aminoglycoside use and genetic factors related to the hearing impairment were identified in all available members of five Chinese pedigrees carrying the A1555G mutation. Comprehensive family and medical histories of probands and other members of these Chinese families showed no other clinical abnormalities, including visual dysfunction, diabetes, muscular diseases, and neurological disorders. All pedigrees had multiple affected individuals (Figure 1). There were 36 individuals with aminoglycoside-induced, non-syndromic bilateral hearing loss in these five pedigrees, including seven individuals from whom no blood samples were obtained (KM001-III-2, KM001-III-14, KM008-III-3, KM011-III-19, KM011-III-20, KM011-IV-7, and KM011-IV-11), one who had died (KM009-II-1), and two who were lacking hearing test results (KM003-II-5 and KM009-II-9). The remaining 26 individuals with aminoglycoside-induced or non-syndromic bilateral hearing loss had different clinical characteristics (Table 1). The age of these 26 individuals ranged from 18 to 52 years. The age of onset of the hearing loss ranged from less than one year to 20 years in 13 patients, while the other 13 individuals or their family members did not remember the specific time of onset. All had a history of receiving aminoglycoside antibiotics, except five individuals who were not certain (Table 1). Meanwhile all normal hearing members carrying the 1555A > G mutation have no history of aminoglycosides usage. Audiological evaluations revealed symmetric hearing impairment in all 26 individuals (Figure 2). However, the subjects exhibited variable severity of the hearing impairment, with two, ten, four, and ten subjects having mild, moderate, severe, and profound hearing impairment, respectively. The subjects also had different audiometric configurations; two subjects had flat patterns, and 24 had slope patterns. The five Chinese pedigrees exhibited different hearing loss penetrance: 52%, 18.2%, 10%, 26.7%, and 44.1% (Table 2), with an average penetrance of 30.2%.

**Table 1:** Summary of clinical data on deafness in five Chinese pedigrees carrying a heteroplasmic A1555G mutation

**Subject**	**Gender**	**Age**	**Audiometric configuration**	**Use of aminoglycosides**	**Age at onset (y)**	**PTA (dB) right ear**	**PTA (dB) left ear**	**Level of hearing impairment**	**Heteroplasmy rate (%)**
KM001-II-2	F	50	Slope	Yes	1	76	70	Severe	93.47
KM001-II-4	F	45	Slope	Yes	Not known	40	51	Mild	91.02
KM001-II-6	F	43	Slope	Yes	Not known	41	41	Moderate	92.01
KM001-II-8	F	41	Slope	Not known	Not known	76	63	Moderate	93.25
KM001-II-10	F	39	Slope	Not known	Not known	40	39	Mild	95.05
KM001-II-13	M	36	Slope	Yes	Not known	54	60	Moderate	95.01
KM001-III-1	F	29	Slope	Yes	1.5	74	74	Severe	94.16
KM001-III-3	F	23	Slope	Yes	2	93	95	Profound	93.60
KM001-III-4	M	24	Slope	Yes	1	95	93	Profound	93.13
KM001-III-5	F	22	Slope	Yes	Not known	43	54	Moderate	91.50
KM001-III-7	F	22	Slope	Yes	12	50	70	Moderate	93.54
KM003-III-4	F	18	Slope	Yes	Not known	103	100	Profound	96.08
KM008-III-8	F	19	Slope	Yes	2	104	109	Profound	66.76
KM009-II-5	F	44	Slope	Yes	Not known	98	76	Severe	94.24
KM009-III-3	M	20	Slope	Yes	Not known	98	95	Profound	94.92
KM011-III-7	M	35	Slope	Yes	6	83	80	Severe	86.23
KM011-III-8	M	30	Flat	Not known	20	59	68	Moderate	87.20
KM011-III-9	M	52	Slope	Yes	3	100	100	Profound	83.58
KM011-III-11	F	45	Slope	Yes	Not known	51	55	Moderate	89.29
KM011-III-15	F	40	Flat	Yes	3	110	110	Profound	82.32
KM011-III-17	F	37	Slope	Yes	4	69	73	Moderate	85.19
KM011-IV-1	F	26	Slope	Not known	Not known	46	49	Moderate	85.87
KM011-IV-2	M	24	Slope	Not known	Not known	51	48	Moderate	91.14
KM011-IV-4	F	20	Slope	Yes	2	109	103	Profound	87.48
KM011-IV-5	F	20	Slope	Yes	Not known	93	93	Profound	94.65
KM011-IV-10	F	20	Flat	Yes	2	94	93	Profound	93.80

**Table 2:** Summary of genetic and molecular data for 5 Chinese families carrying the heteroplasmy A1555G mutation

**Pedigree**	**Number of matrilineal relatives**	**Number of patients**	**Penetrance**^**a **^**(%)**	**Generation**	**Number of generation**	**Heteroplasmy level**^**b **^**(%)**			
						**Min**	**Max**	**Generation mean**	**Pedigree mean**
KM001	25	13	52	II	7	91.02	95.05	93.19	93.34
				III	11	91.50	94.16	93.43	
KM003	11	2	18.2	II	3	32.87	94.31	69.76	78.28
				III	4	58.69	96.08	84.68	
KM008	20	2	10	I	1	23.55	23.55	23.55	31.98
				II	5	0	43.79	26.63	
				III	9	0	91.35	35.89	
KM009	15	4	26.7	I	1	91.38	91.38	91.38	93.57
				II	3	92.21	94.24	93.41	
				III	2	94.41	94.92	94.67	
KM011	34	15	44.1	II	2	87.85	88.69	88.27	87.99
				III	12	82.32	90.89	86.78	
				IV	6	85.87	94.65	90.31	

^a^Affected matrilineal relatives/total matrilineal relatives.

^b^Area of G allele/(area of A allele + area of G allele).

### Mutational screening of the 12S rRNA gene in maternal subjects

**Figure 3:**
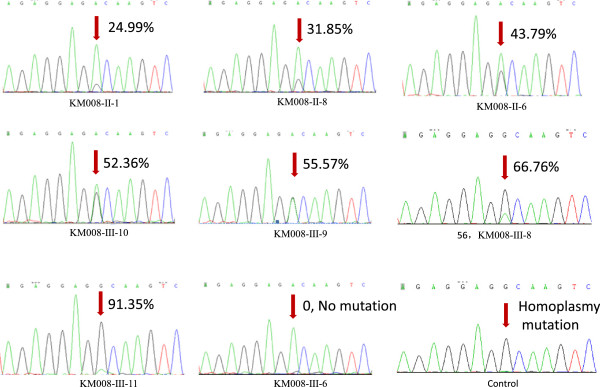
**Direct sequencing analysis of the A1555G.** Peaks arrow denoted represented wild-type allele and mutation-type allele at 1555 loci with different heteroplasmy level. The heteroplasmy level were marked aside the arrow.

To elucidate the molecular basis of the hearing loss, we initially performed a mutational analysis of the mitochondrial 12S rRNA gene in all affected maternally related individuals in the five pedigrees who were seen in the Department of Otolaryngology of the Chinese PLA General Hospital. Initially, 66 individuals were detected. DNA spanning the 12S rRNA from each subject was PCR amplified and sequenced. Sixty-two individuals harbored a heteroplasmic mtDNA A1555G mutation in the 12S rRNA gene, with different relative sizes of the A and G peaks (Figure 3). The other four individuals appeared to have no A1555G mutation (KM008-II-3, KM008-III-5, KM008-III-6, and KM008-III-7), as we did not find any G mutation peak in the direct sequencing graphs of these four individuals. However, if the heteroplasmy rate was too high or too low, we would not have been able to distinguish homoplasmy, heteroplasmy, or wild type using standard Sanger sequencing technology.

### Mutational analysis of GJB2

To examine the role of GJB2 gene in the phenotypic expression of the A1555G mutation, we performed the mutational screening of GJB2 gene in probands of these five Chinese pedigrees. No GJB2 mutation was found. Indeed, the absence of a GJB2 gene mutation in these hearing impaired subjects suggested that the GJB2 gene might not modify the phenotypic effects of the A1555G mutation in them.

### Analysis of the heteroplasmy levels of family members

To identify the A1555G mutation and explore the heteroplasmy inheritance pattern, we used the SNaPshot technique to quantify the heteroplasmy level. The average heteroplasmy rates differed among the five pedigrees (Table 2, Figures 1 and 3). The average heteroplasmy rate of the KM001 pedigree was 93.34% (range, 91.02–95.05%); the average heteroplasmy rates of generations II and III in the KM001 pedigree were 91.19% and 91.43%, respectively. The average heteroplasmy rate of the KM003 pedigree was 78.28% (range, 32.87–96.08%), with average heteroplasmy rates of 69.76% and 84.68% in generations II and III, respectively. For the KM008 pedigree, the average heteroplasmy rate was 31.98% (range, 0–91.35%), and the average heteroplasmy rates of generations I to III were 23.55%, 26.63%, and 35.89%, respectively. The KM009 pedigree had an average heteroplasmy rate of 93.57% (range, 91.38–94.24%); the average heteroplasmy rates of generations I to III were 91.38%, 93.41%, and 94.67%, respectively. The average heteroplasmy rate of the KM011 pedigree was 87.99% (range, 82.32–94.65%), and the average heteroplasmy rates of generations II to IV were 88.27%, 86.78%, and 90.31%, respectively.

The KM008-III pedigree included four members (KM008-II-3, KM008-III-5, KM008-III-6, and KM008-III-7) with no A1555G mutation based on direct sequencing. The mother of KM008-II-3 was KM008-I-2, whose mtDNA A1555G heteroplasmy rate was 23.55%. The heteroplasmy rates of the brothers and sisters of KM008-II-3 ranged from 24.99–3.79%. The offspring of KM008-II-3 (KM008-III-5, KM008-III-6, and KM008-III-7) had no mtDNA A1555G mutation, like their mother (KM008-II-3), and they all had normal hearing. We did not find any relationship that could explain the increases or decreases in the mtDNA A1555G heteroplasmy levels of the different generations of these five pedigrees.

### Clinical and genetic evaluation of the five pedigrees with the A1555G mutation

To explore the relationship between the clinical features and heteroplasmy levels in the pedigrees, the hearing loss levels of five probands in the five pedigrees, including KM001-III-4, KM003-III-4, KM008-III-8, KM009-III-3, and KM011-IV-5, and their heteroplasmy levels were analyzed. All five probands had symmetric, profound, slope pattern hearing impairment (Table 1 and Figure 2). Their respective heteroplasmy levels were 93.13, 96.08, 66.76, 94.92, and 94.65%, and the average heteroplasmy levels of the pedigrees were 93.34, 78.28, 31.98, 93.57, and 87.99%, respectively (Table 2).

Among individuals, two had mild hearing loss, the average age of them was 42 years, their heteroplasmy levels were 91.02% and 95.05%, with an average of 93.04%. Ten had moderate hearing loss, the average age of these members was 32.6 years, their heteroplasmy levels ranged from 85.19% to 95.01% and averaged 90.36%. Four had severe hearing loss, the average age was 39.5 years, with heteroplasmy levels of 93.47, 94.16, 94.24, and 86.23%, and an average of 92.05%. Another 10 individuals had profound hearing loss, the average age of these 10 members was 25.6 years, their heteroplasmy levels ranged from 66.76% to 96.08% and averaged 88.63%. No regular pattern was identified (Table 1).

We also analyzed the percentage of deafness in all maternally related members according to the heteroplasmy level (Figure 1). In our pedigrees, four members lacked the mtDNA A1555G mutation (KM008-II-3, KM008-III-5, KM008-III-6, and KM008-III-7); all had normal hearing. Eight members had heteroplasmy levels of <50% (KM003-II-7, KM008-I-2, KM008-II-1, KM008-II-5, KM008-II-6, KM008-II-8, KM008-III-4, and KM008-III-12); they all also had normal hearing. Another four had heteroplasmy levels from 50% to 80% (KM003-III-3, KM008-III-8 KM008-III-9, and KM008-III-10). Only one (KM008-III-8, with a heteroplasmy level of 66.76%) was deaf, giving a 25% risk for deafness associated with this heteroplasmy level. Seventeen members in the five pedigrees had heteroplasmy levels of 80–90%, eight of whom had deafness for a risk of 47.06%. The heteroplasmy level of the remaining 33 members exceeded 90%, and 19 had deafness, for a risk of 57.58%. Thus, the risk for deafness tended to increase with an increase in the heteroplasmy level.

## Discussion

This study examined the clinical, genetic, and molecular characteristics of five Chinese pedigrees with aminoglycoside-induced, non-syndromic hearing impairment associated with the heteroplasmic A1555G mutation and analyzed the levels and inheritance of heteroplasmy for this mutation.

Hearing impairment as the sole clinical phenotype was present only in the maternal lineages of these pedigrees with the A1555G mutation. The hearing impairment phenotype and penetrance varied among the pedigrees, as reported for other pedigrees [5-9]. In our subjects, the onset age of hearing impairment ranged from younger than 1 year old to 20 years old. The onset of hearing loss was related to the use of aminoglycosides.

The direct sequencing mutation analysis initially revealed the heteroplasmy trait of the A1555G mutation in probands and their pedigrees (Figure 3). However, not all individuals showed heteroplasmy by direct sequencing because the mixed peak influenced the results, especially when the heteroplasmy was very low or high. To accurately determine the heteroplasmy level, we used the SNaPshot technique to quantify the heteroplasmy level. This technique is used specifically to genotype single nucleotide polymorphisms (SNPs). According to previous studies, the SNaPshot technique is an accurate, sensitive, flexible, and cost-effective method for examining many mutations or SNPs in one run of the SNaPshot assay [16,17]. It is also an accurate, reproducible, and sensitive method for determining heteroplasmic mtDNA mutations in different tissues, and it is a promising system for use in the pre- and postnatal diagnosis of mtDNA-associated disorders [18]. In the present study, the heteroplasmy level could be calculated reliably using the SNaPshot technique, which provides a technical platform for the quantitative detection of the mtDNA A1555G mutation.

We identified variable transmission of heteroplasmy across different generations in the five Chinese pedigrees. Within each pedigree, the heteroplasmy level differed among individuals and among generations. This may be explained by the peculiarities of mtDNA, i.e., different mutation loads can occur in different family members with mitotic segregation in a cell [19]. Although the average heteroplasmy tended to increase in generations, except generation III of KM011, there was no observed tendency for an increase or decrease across individuals, not only from mothers to children but also in siblings. For example, the heteroplasmy level of KM003-II-1 was 82.10%, that of her son KM003-III-2 was 90.16%, and that of the other son was 58.69% (Figure 1). In the KM008 pedigree, four members (KM008-II-3, KM008-III-5, KM008-III-6, and KM008-III-7) had no mutation at all. The mother of KM008-II-3 was KM008-I-2, whose heteroplasmy rate was 23.55%. KM008-II-3 did not inherit any A1555G mutation, and all her offspring lacked the mtDNA A1555G mutation (KM008-III-5, KM008-III-6, and KM008-III-7) and had normal hearing. This phenomenon may be attributable to the so-called “mitochondrial bottleneck” and “random allocation” [20,21], meaning that a small number of mutated mtDNA molecules become the founders for the offspring’s mtDNA when mitosis takes place. When the number of segregating units (groups of clonal mtDNA that co-segregate) that become the mtDNA founders of the embryo is small, large fluctuations in heteroplasmy may occur in a single generation.

To explore the relationship between clinical features and genetic backgrounds, we analyzed the auditory threshold of hearing loss and heteroplasmy level of the mtDNA A1555G mutation within pedigrees and among members, as well as the percentage of all maternally related members with deafness according to different heteroplasmy levels. The analysis indicated that the risk for deafness increased with an increase in the heteroplasmy level. The risk for deafness was 0 for members with heteroplasmy levels <50%, 25% for members with heteroplasmy levels from 50–80%, 47.06% for members with heteroplasmy levels from 80–90%, and 57.58% for members with heteroplasmy levels exceeding 90%. In this heredity mitochondrial trait, there was a threshold effect regarding the onset of disease. In our study, all subjects with heteroplasmy levels <50% had normal hearing, and 25% of the subjects with heteroplasmy levels between 50–80% had hearing loss. This implies that the heteroplasmy level of the mtDNA A1555G mutation must exceed 50% to cause the hearing-loss phenotype. Further functional studies should confirm this. Given that the risk for deafness increased with the heteroplasmy level, the heteroplasmy level may influence the penetrance of deafness related to the mtDNA A1555G mutation, as seen with other factors, including ethnic background, environment, aminoglycoside use, nuclear genes, mitochondrial haplotypes/variants, and the threshold effect [5-9].

Based on our data, the homoplasmy variation, including that for the A1555G mutation, is transmitted stably in all maternal lineages of a pedigree based on mitochondrial maternal hereditary; however, the heteroplasmy variation is likely to involve more complicated genetic and clinical factors. The risk for deafness increased with the heteroplasmy level, which also appeared to influence the penetrance of deafness associated with the mtDNA A1555G mutation.

## Conclusions

There were large, seemingly random differences in the heteroplasmy level between mothers and offspring in the process of maternal transmission. The heteroplasmy could disappear when the heteroplasmy level of pedigree was sufficiently low. No regular pattern was identified, other than the tendency for an increased risk for deafness with an increase in the heteroplasmy level. The heteroplasmy level may also influence the penetrance of deafness related to the mtDNA A1555G mutation.

## Competing interests

The authors declare that they have no competing interests.

## Authors’ contributions

Conceived and designed the experiments: PD YHZ SQZ. Performed the experiments: YHZ DYK MYH GJW YYY YS. Analyzed the data: YHZ SSH HJY SQZ PD. Contributed reagents/materials/analysis tools: YHZ SSH. Wrote the paper: YHZ SSH PD. All authors read and approve the final manuscript.

## Authors’ information

Yuhua Zhu and Shasha Huang as co-first authors.
